# Retrograde pyeloperfusion: a novel technique to prevent stone fragment migration into the ureter during percutaneous nephrolithotomy

**DOI:** 10.1007/s00240-025-01854-6

**Published:** 2025-10-15

**Authors:** Adel Battikha, Ali Albaghli, Kallan Richards, Ninous Betdashtoo, Faith Ajayi, Jongwan Park, Elizabeth A. Baldwin, Grant Sajdak, Ruben Crew, Daniel Jhang, Zham Okhunov, D. Duane Baldwin

**Affiliations:** 1 Department of Urology, Loma Linda University, Loma Linda, CA USA; 2 Department of Urology, Loma Linda University Health, 11234 Anderson Street, Loma Linda, CA 92354 USA

**Keywords:** Percutaneous nephrolithotomy, Kidney stone, Ureteroscopy

## Abstract

To determine whether retrograde pyeloperfusion (RP) during percutaneous nephrolithotomy (PCNL) decreases antegrade ureteral stone fragment migration, normal, moderately, and severely hydronephrotic silicone kidney and ureter models were created using deidentified CT images. Two techniques to prevent fragment migration, ureteral access sheath (UAS) and RP via a ureteroscope, were compared to an empty ureter. Two grams of stone fragments were placed into the renal pelvis. Antegrade irrigation and suction were applied for 5 min. The weights of ureteral stone fragments were compared using the Kruskal-Wallis test (*p* < 0.05 significant). In the normal ureter model, RP significantly reduced ureteral stone fragment migration (0.06±0.03 g) compared to both the UAS (0.32±0.19 g; *p* = 0.047) and control (0.42±0.11 g; *p* = 0.009). Similarly, in the moderate hydroureter model, RP significantly reduced ureteral stone fragment migration (0.11±0.06 g) compared to both the UAS (0.68±0.18 g; *p* = 0.009) and control (0.48±0.15 g; *p* = 0.016). In the severe hydroureter model, RP reduced ureteral stone fragment migration (0.17±0.12 g) compared to the UAS (1.04±0.29 g; *p* = 0.026) but was similar to the control (0.65±0.37 g; *p* = 0.169). RP showed an 85.7% and 81.1% reduction compared to the control and UAS conditions in the normal model, 76.2% and 83.3% reduction in the moderate hydroureter model, and 73.3% and 83.2% reduction in the severe hydroureter model, respectively. Thus, compared to an empty ureter and a UAS, in all ureter models, RP reduced ureteral stone fragment migration by 73.3–85.7%. Retrograde pyeloperfusion should be considered to reduce ureteral stone fragment migration during PCNL when endoscopic combined intrarenal surgery is employed.

## Introduction

Percutaneous nephrolithotomy (PCNL) is the first-line treatment for complex renal calculi, including stones larger than 20 mm and staghorn stones [1]. In the process of stone fragmentation during PCNL, fragments may migrate, becoming entrapped in the ureter. This process is often exacerbated by the positive pressure gradient created by nephroscope antegrade irrigation. Stone fragment migration and obstruction hinders clearance rates and increases risk of post-operative complications including kidney obstruction, infection, renal colic, and growth of stone fragments requiring retreatment [2–5].

Many devices have been adapted to perform retrograde irrigation through the ureter to reduce stone migration. Bilen et al. achieved retrograde irrigation with a ureteral catheter in the setting of a minimally invasive PCNL (mini-PCNL), which improved the removal of small stone fragments present within the collecting system [6]. Additionally, retrograde irrigation through ureteral stents has been shown to improve surgical field visibility, decrease operation length, improve stone clearance, and decrease bleeding during PCNL [7]. Furthermore, retrograde irrigation through a flexible ureteroscope performed simultaneously with antegrade irrigation during mini-PCNL improved the wash out of small stone fragments, resulting in reduced residual stones [8]. However, no study has examined using retrograde irrigation by flexible ureteroscope during standard PCNL.

The aim of this study was to compare ureteral stone fragment migration between control (empty ureter), ureteral access sheath (UAS), and retrograde pyeloperfusion (RP) in a benchtop PCNL model.

## Materials and methods

### Preparation of a kidney model

Using 3D Slicer version 5.2.2 [9], (Kitware, New York, USA), a kidney and collecting system mold were created based on CT scans of a deidentified patient. The mold was fabricated with an Ultimaker 3 Extended 3D printer (Ultimaker, Geldermalsen, Netherlands) using polylactic acid (3D Universe, Chicago, IL). The mold was filled with liquid Dragon Skin™ 20 silicone, (Smooth-On, Inc., Macungie, PA), and allowed to cure for six hours before use. A 30 Fr inner diameter Amplatz Type Renal Sheath, (Boston Scientific, Marlborough, MA), was inserted into the posterior upper calyx of the model to allow for direct passage of the instrumentation into the collecting system of the kidneys.

### Preparation of ureter models

 Autodesk Fusion 360 (Autodesk, San Francisco, CA) was used to design a physiologically accurate ureter based on dimensions obtained from the literature [10–13]. The diameter of the normal ureter model was scaled up to create moderate and severe hydroureter models (Table 1). The ureter molds were fabricated in the same manner as the kidney models. Each mold was coated with Dragon Skin™ 20 silicone and allowed to cure for 2 h; this process was repeated with the ureter oriented in the opposite vertical direction to obtain a smooth, thick coating of silicone on the surface of the mold.

**Table 1:**
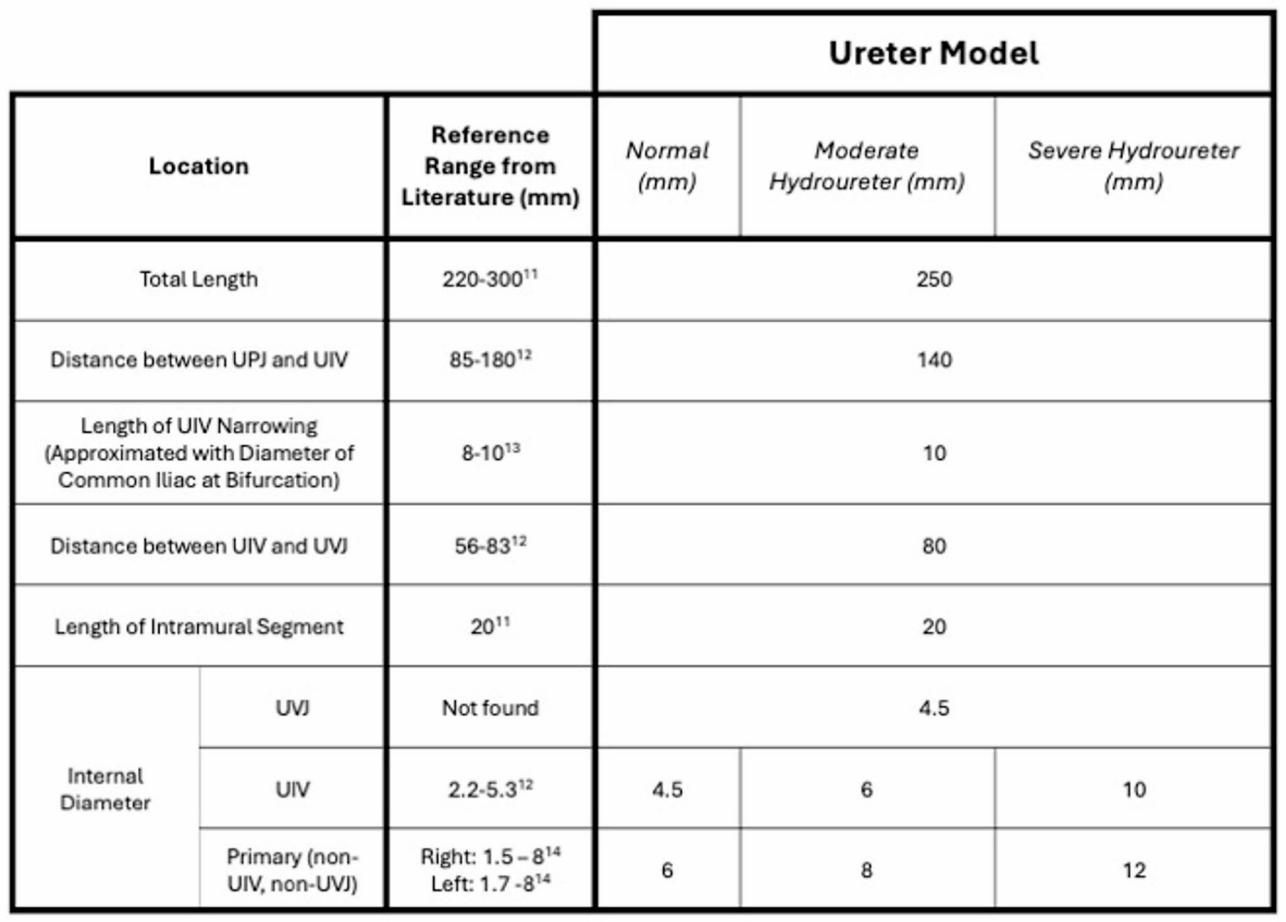
Literature-based ureter dimensions to obtain a normal ureter model. Dimensions adjusted to obtain moderate and severe hydroureter models

### Preparation of kidney stone fragments

A 15:3 BegoStone to water ratio was utilized to simulate calcium oxalate monohydrate consistency kidney stones [14]. The stones were allowed to dry before being crushed into smaller fragments. The fragments were filtered through two sieves with pore diameters of 1- and 2-mm; fragments that passed through the 2-mm sieve were then applied to the 1-mm sieve. Only fragments that did not pass through the 1-mm sieve were collected, leaving stone sizes of 1–2 mm.

### Experimental set-up

**Fig. 1:**
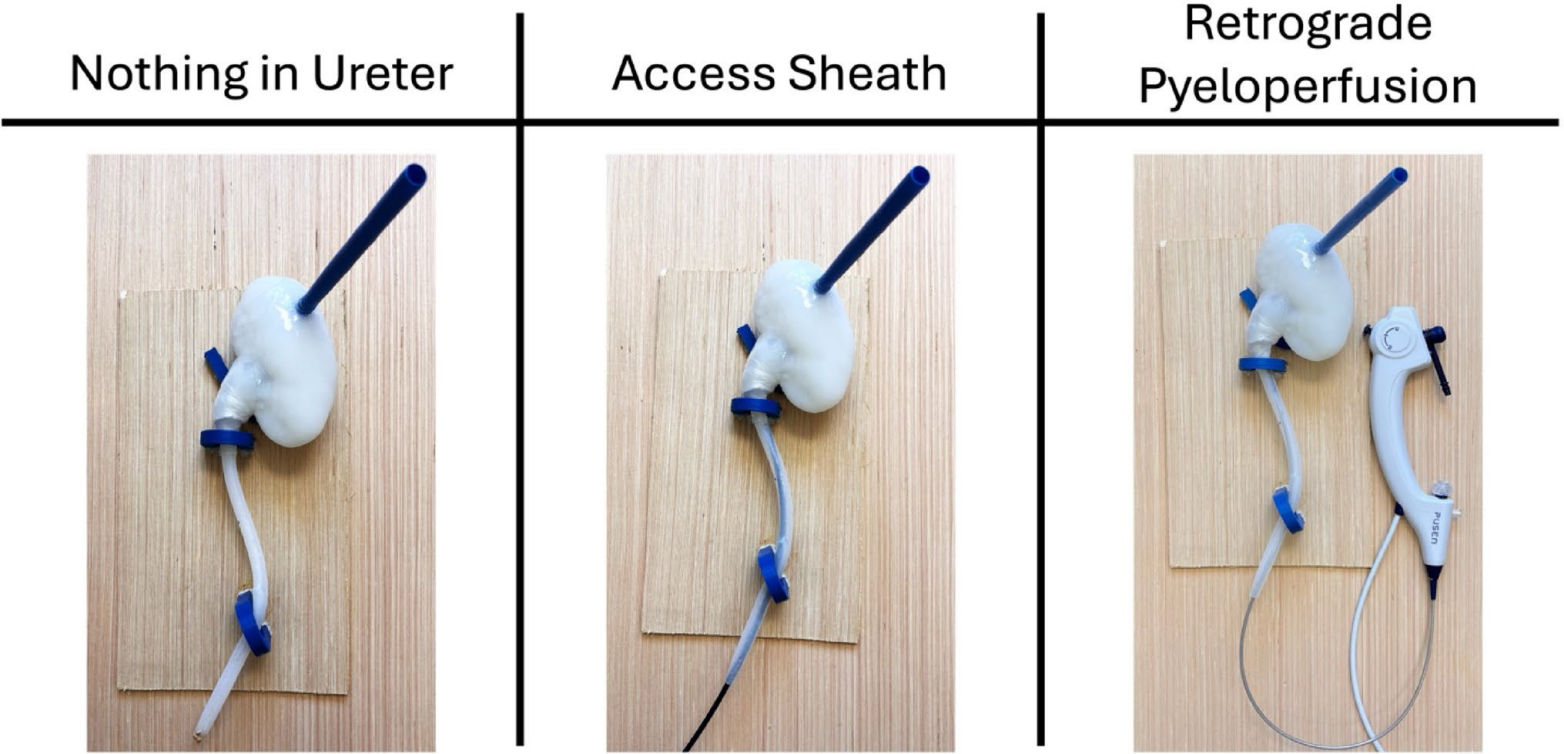
Experimental set-up for each of the following conditions: nothing in ureter (control), access sheath in ureter, and RP with flexible ureteroscope in ureter

To evaluate the effect of RP in different degrees of hydroureter, 2.00 g of 1–2 mm stone fragments were placed into the renal pelvis. A ureter was then attached to the renal pelvis, and the interface was surrounded by a layer of Parafilm (Uline, Pleasant Prairie, WI) to prevent fluid leakage. The kidney and ureter models were secured into a scaffold that mimicked the natural path of the ureter in the body, securing the ureter into place at the ureteropelvic junction (UPJ) and at the crossing of the iliac vessels. For each ureter model, two experimental conditions, a 10/12 Fr UAS (Cook Medical, Bloomington, IN) and RP via 7.5 Fr flexible ureteroscope (Pusen Medical, Zhuhai, China), were compared to control (empty ureter). Insertion of the UAS and the ureteroscope in the silicone ureterovesical junction (UVJ) created resistance of the intramural ureter, while a loose rubber compression band at the UVJ was used in the control group (Fig. 1).

Antegrade irrigation was employed in all experimental conditions by using intravenous (IV) tubes running a 1000 cc water bag at a standardized height of 2.3 m on an IV pole, connected to either a 25 Fr rigid nephroscope (Olympus, Tokyo, Japan), or a 16 Fr flexible nephroscope (KARL STORZ, Tuttlingen, Germany). The 30 Fr renal access sheath was positioned with the tip located in the posterior upper pole calyx. The rigid nephroscope and the flexible nephroscope were both positioned in the renal pelvis alternately. The rigid nephroscope was gently oscillated and the flexible nephroscope was deflected to visualize the renal calyces. RP was achieved in a similar manner with the water bag connected to a flexible ureteroscope, with its tip advanced to the ureteropelvic junction (UPJ). For all trials involving a UAS, the tip of the sheath was advanced to a point 2 cm distal to the UPJ.

### Experimental trials and data collection

 Each trial was run for 5 min with alternating periods of 35 s of gravity irrigation with the rigid nephroscope, 5 s of suction with a 3.6 mm shock-pulse ultrasound probe (Olympus Corporation, Tokyo, Japan) connected to the Neptune suction unit (Stryker, Kalamazoo, MI), and 35 s of irrigation with the flexible nephroscope. This was done to mimic the use of the rigid nephoscope for stone fragmentation and the use of the flexible nephoscope for renal mapping. All instrumentations were then removed from the kidney model and the ureter was detached. Ureteral stone fragments were collected, dried for 24 h, and weighed.

### Statistical analysis

Statistical analysis was performed using Jamovi Version 2.5.7 (The Jamovi Project, Sydney, Australia). The Kruskal-Wallis test with post-hoc analysis was employed to identify significant differences between groups (*p* < 0.05 significant).

## Results

**Fig. 2:**
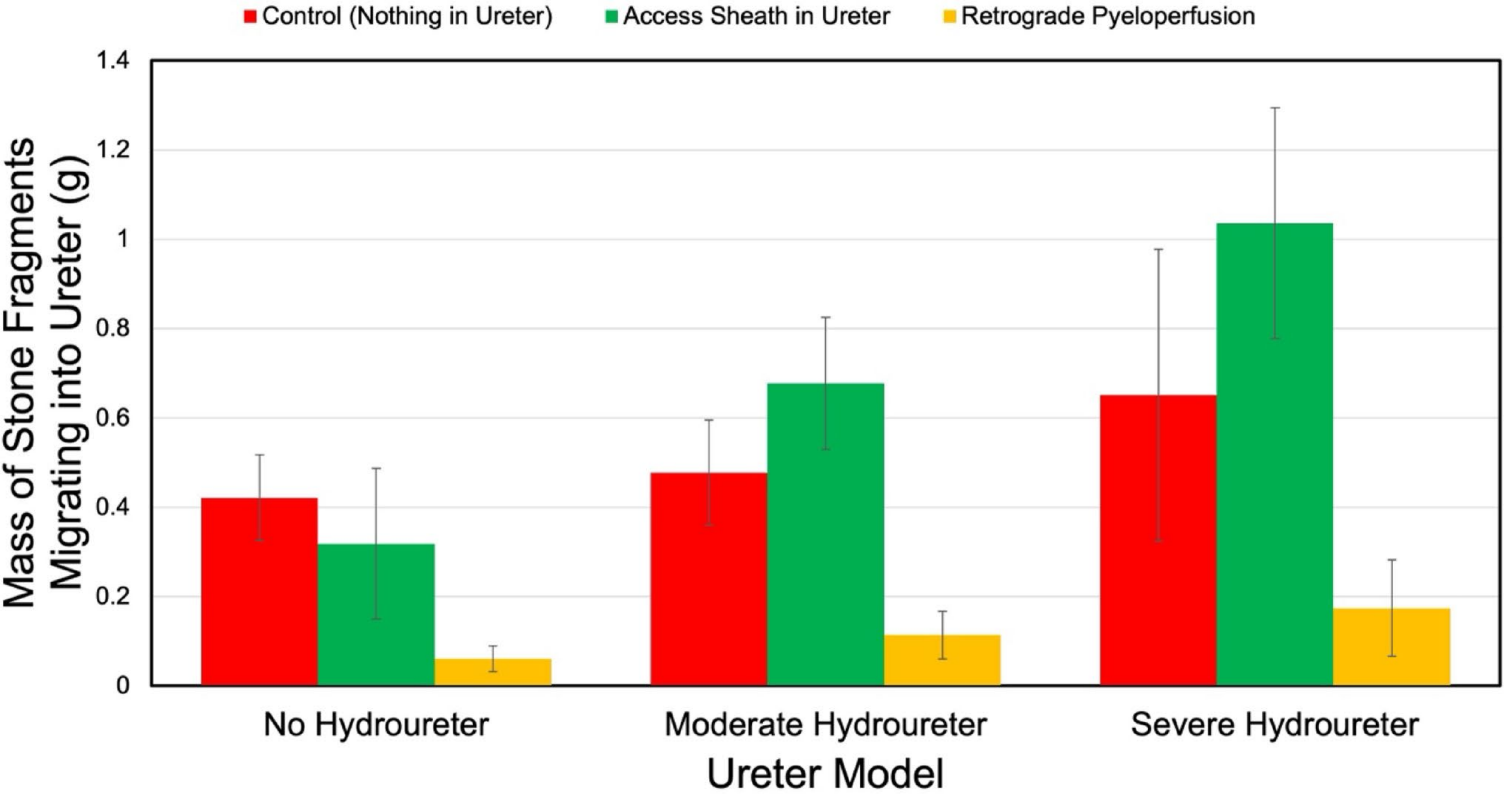
Mass of stone fragments remaining in normal, moderate, and severe hydroureter models for each experimental condition. Error bars represent standard error

In all ureter models tested, RP demonstrated decreased ureteral stone fragments relative to the control and UAS **(**Fig. 2). In the normal ureter model, the mass of migrated stone fragments was 0.42±0.11 g for the control group, 0.32±0.19 g for the UAS group, and 0.06±0.03 g for the RP group **(**Table 2). Statistical analysis showed that RP had significantly less ureteral stone fragments than the control (*p* = 0.009) and the UAS (*p* = 0.047), with no significant difference between the control and UAS groups (*p* = 0.175). In the moderate hydroureter model, the migrated stone fragments were 0.48±0.15 g for the control, 0.68±0.18 g for the UAS, and 0.11±0.06 g for the RP. RP again showed significantly less stone migration compared to both the control (*p* = 0.016) and the UAS (*p* = 0.009), with no significant difference between the control and UAS groups (*p* = 0.347). In the severe hydroureter model, the migrated stone fragments were 0.65±0.37 g for the control, 1.04±0.29 g for the UAS, and 0.17±0.12 g for the RP. In this model, although RP had less ureteral stone fragments than the control, it did not reach significance (*p* = 0.169); RP had significantly less ureteral stone fragments than UAS (*p* = 0.026).

**Table 2:** Mass (g) (±SD) of Phantom kidney stone fragments remaining in different ureter models following a specified trial period

Ureter Size	Control	Ureteral Access Sheath (UAS)	Retrograde Pyeloperfusion (RP)	*p*-value		
				Control vs. UAS	Control vs. RP	RP vs. UAS
Normal	0.42 (0.11)	0.32 (0.19)	0.06 (0.03)	0.175	0.009*	0.047*
Moderate	0.48 (0.15)	0.68 (0.18)	0.11 (0.06)	0.347	0.016*	0.009*
Severe	0.65 (0.37)	1.04 (0.29)	0.17 (0.12)	0.465	0.169	0.026*

Bolded values with asterisk denotes significance

To further quantify the effectiveness of RP, we calculated the percent reduction in ureteral stone mass compared to the control and UAS conditions. In the normal ureter model, RP demonstrated an 85.7% and 81.1% reduction in ureteral stone mass relative to the control and UAS conditions, respectively. In the moderate hydroureter model, RP reduced ureteral stone mass by 76.2% and 83.3% relative to the control and UAS conditions, respectively. In the severe hydroureter model, RP decreased ureteral stone mass by 73.3% and 83.2% when compared to the control and UAS conditions, respectively.

When data from all three ureter models were aggregated, the average ureteral stone fragment masses were 0.52 g (range: 0.084–1.906 g) for the control group, 0.68 g (range: 0.009–1.376 g) for the UAS group, and 0.116 g (range: 0–0.493 g) for the RP group. RP also demonstrated significantly less fragment migration than both the control and UAS groups (*p* < 0.001 for both), which represented a 77.6% and 82.9% reduction, respectively. No significant difference was observed between the control and UAS groups (*p* = 0.52).

**Fig. 3:**
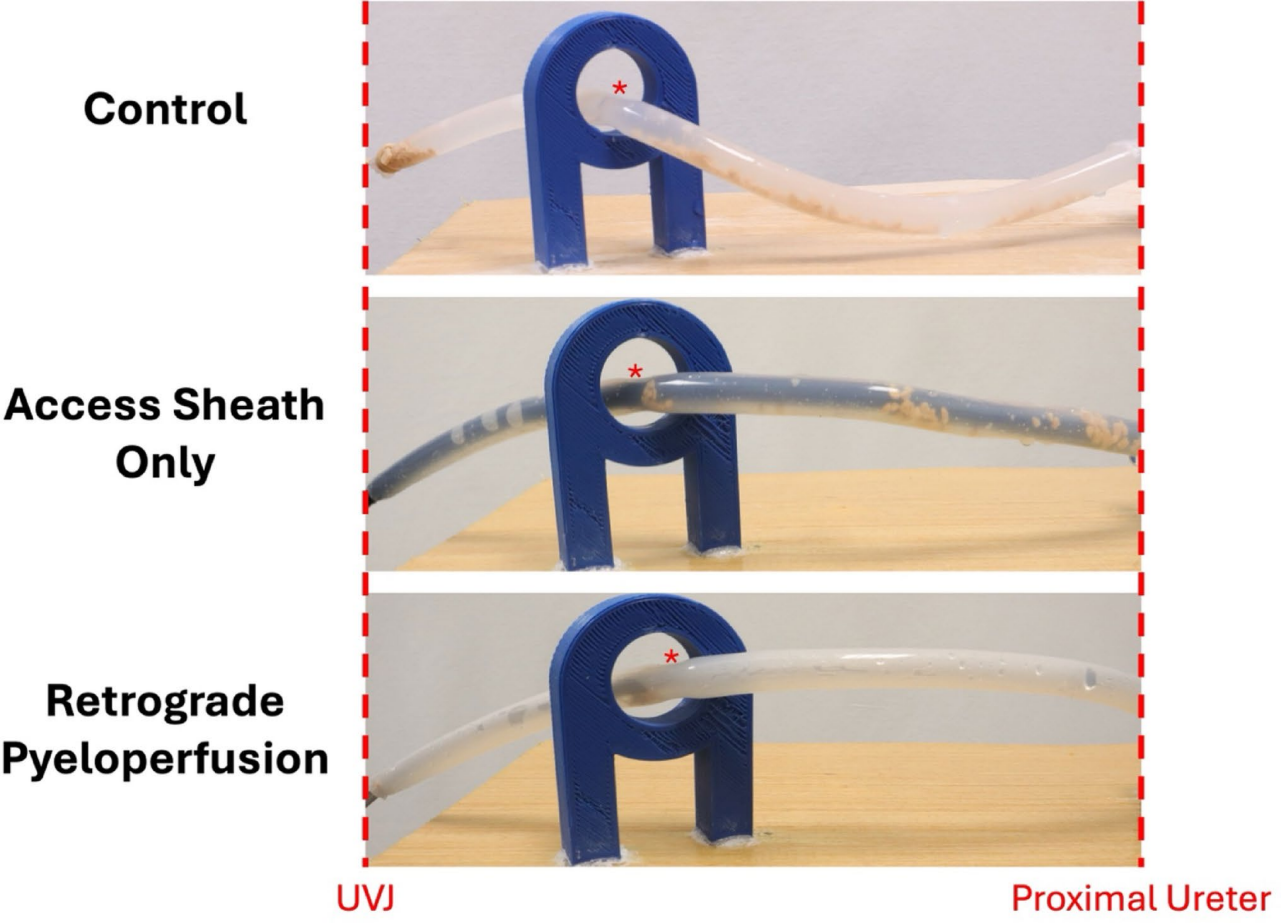
Demonstration of amount and location of stone fragments in a normal ureter model with each experimental condition. UVJ = ureterovesical junction, * = location of ureteral crossing of iliac vessels (UIV)

As seen in Fig. 3, RP decreased stone fragment migration relative to the control and UAS conditions. In the normal ureter model, there was significant stone migration to the distal portions of the ureter when the ureter was empty. With the UAS in place, the stone fragments migrated less, but many fragments traveled down to the narrowing created by the ureter crossing the iliac vessels. However, with RP, fewer stone fragments reached the distal portions of the ureter and the physiologic narrowing created by the iliac crossing, compared to the other experimental conditions.

## Discussion

Ureteral stone fragment migration during PCNL can add significant risk for complications, re-intervention, and lengthen operative time. In this study, two strategies (RP and UAS) for preventing ureteral stone fragment migration were compared to an empty ureter, which served as the control. RP significantly reduced stone fragment migration into the ureter compared to an empty ureter and UAS for both the normal ureter and moderate hydroureter models. It was demonstrated that RP resulted in at least a 76% decrease in stone migration in the non-dilated and moderate hydroureter models compared to both control and UAS groups. In the severe hydroureter model, although not statistically significant, RP reduced ureteral stone fragments by 73% compared to control. Compared to the control group, UAS showed an acceptable reduction (24%) in stone migration in the non-dilated hydroureter.

Stone migration to the ureter during PCNL remains a significant clinical challenge [15]. This issue is frequently encountered and poses the risk of ureteral obstruction, which is associated with complications such as urosepsis, acute kidney injury, persistent postoperative pain and low stone free rates [16]. In addition, operative time may be extended as efforts are made to retrieve the fragments and fully clear the ureter. Depending on the patient and stone characteristics, the fragments may pass spontaneously. However, if fragments fail to pass, patients may be subjected to additional procedures or emergent hospital visits. Left untreated, stone fragments can increase in size, making it imperative to attempt complete fragment removal during the initial procedure [17].

Multiple techniques and instruments have been developed and implemented to reduce the risk of stone fragment migration into the ureter during PCNL, ranging from modified procedural approaches to the use of specialized anti-migration devices [17]. The use of larger caliber or multiple accesses has been reported to reduce the pressure gradient forcing stone fragments down the ureter but can lead to additional complications such as bleeding and decline in renal function [17].

Anti-migration devices have generated a considerable interest as a solution to this problem. While originally designed to prevent stone retropulsion in ureteroscopy, various anti-migration devices have been adopted and employed in PCNL [18]. One such device is the occlusive balloon catheter, which is placed via cystoscopy under fluoroscopic guidance to obstruct the ureter distal to the stone, preventing migration of fragments during lithotripsy. Despite its success, its use is limited in dilated ureters, as they are unable to effectively occlude the entire ureter diameter. Moreover, concerns regarding increased intrarenal pressure and potential ureteral mucosal injury during prolonged cases have been raised [19]. Another device, the Stone Cone (Boston Scientific Microvasive, Natick, MA, USA), is a helical device that is made from a stainless-steel spiral wand, with nitinol strand coated with polytetrafluoroethylene (PTFE). The device can be deployed during the renal access, then excluded from the working sheath by using an additional guidewire for access dilation, allowing for flexibility during PCNL. Its unique design prevents stone fragments ≥ 2 mm from migrating while maintaining low intrarenal pressure. However, its design makes it prone to damage the ureteral mucosa during tract dilation or stone fragmentation and increases procedural costs [20].

Other innovative solutions have been proposed. Reversable thermosensitive polymer gels, such as BackStop (Boston Scientific Inc. Customer Service, Natick, MA), are injectable, water-soluble, and biocompatible materials that condense to create a ureteral barrier when exposed to body temperature. Administered into the proximal ureter under direct vision, the gel creates a 2–4 cm proximal ureteral plug that lasts up to 45 min before naturally breaking down, with the option of reversal through cold water infusion. Studies have reported minimal stone fragment migration into the ureter during PCNL with no cases requiring additional intervention or sustaining any complications [21]. However, polymer gels have limitations. First, due to their transparent nature, it is difficult to confirm proper deployment under vision or using fluoroscopy. Second, during stone fragmentation, the debris can obscure the UPJ, giving the impression that no fragments are passing downwards. Finally, many PCNLS last longer than 45 min, making it challenging to re-inject the material while keeping all fragments confined to the renal pelvis [21, 22].

With advancements in ureteroscopy technology, endoscopic combined intrarenal surgery (ECIRS) is becoming increasingly popular in the management of complex renal stones. ECIRS is an approach that combines both antegrade and retrograde nephroureteroscopy to maximize complex stone treatment. Improvement of both intraoperative efficiency and postoperative outcomes were reported including improved renal access, intraoperative vision, shorter operative time, and increased stone free rate [23]. Since the ureteroscope is already positioned at the UPJ, our institution has employed a technique that utilizes continuous retrograde irrigation through the ureteroscope to prevent stone fragment migration into the ureter. Our initial observations noted that significantly less fragments appeared to migrate into the ureter. Given the wide variety of factors in a clinical setting, we sought to create a benchtop model where these factors can be controlled.

This method for preventing stone fragment migration is particularly applicable for surgeons who employ an ECIRS approach as it requires no additional equipment and is highly effective. Our benchtop study results confirm our clinical experience that RP is highly effective in preventing stone fragment migration. The superior performance of RP can be attributed to the occlusion created by the ureteroscope tip, which prevents fragments from passing beyond its placement, along with the hydrodynamic barrier formed by the continuous retrograde irrigation flow. However, in the severely dilated ureteral model, RP showed a slight limitation, with some fragments still passing beyond the ureteroscope.

In contrast, the UAS demonstrated an acceptable level of stone migration in non-dilated and moderate hydroureter models. The increased stone migration with the UAS in the severely dilated model can be explained by the mismatch between the UAS size and the dilated ureteral diameter. Specifically, the 12 Fr UAS (4 mm) did not completely obstruct the dilated ureter (8 to 12 mm), enabling the fragments to migrate distally along the sheath. Additionally, based on the Bernoulli principle, a given water volume will have a higher velocity passing from a wide area like the dilated renal pelvis, to a narrow area like the UPJ, hence a higher drag of stone fragments down the ureter can be expected [24].

A thorough understanding of the PCNL equipment used during the procedure is crucial. Lithotripsy technologies play a key role in stone removal, and proper settings are essential to control fragmentation and prevent migration. For example, using a single pulse setting in pneumatic lithoclasts has been shown to produce controlled fragmentation with less scatter compared to the multi-pulse setting [25]. Similarly, laser settings are pivotal in determining the strategy for stone fragmentation. High pulse energy increases the stone fragmentation rate, particularly for hard stones, but can also lead to stone relocation if fragmentation occurs outside a confined space, such as a calyx. Therefore, judicious use of laser energy settings is vital to limit fragment migration. Another important laser parameter is pulse duration. Bader et al. examined the impact of short versus long pulses and found that longer pulse settings resulted in smaller fragments, less stone movement, and better overall energy utilization [26, 27].

The use of UAS is a common practice to maintain a stone-free ureter during PCNL; however, these techniques have limitations. When retrograde ureteroscopy is not used, the final ureteral clearance is achieved via antegrade proximal ureteroscopy, performed after the UAS is removed. This method involves pushing the nephroscope as deep as possible into the ureter until resistance is felt, but it is both unsafe and unreliable for ensuring that no fragments remain in the distal ureter. Furthermore, while UAS can reduce intrarenal pressure, it also hinders visualization during the procedure. The blind deployment of UAS increases the risk of ureteral tears, and prolonged use may cause ureteral mucosal vascular compromise, potentially leading to strictures [28, 29].

This study has several limitations. First, the in vitro silicon kidney and ureteral models are not perfect representations of the human renal collecting system, even when simulating different degrees of hydroureter. Second, the study primarily focuses on stone migration prevention and due to its bench-top design, does not assess long-term outcomes such as postoperative complications, stone-free rates, or patient-reported outcomes, which are crucial for evaluating clinical relevance. Third, in the setting of severe hydroureter, exploring different ureteroscopes with different sizes is warranted to optimize the efficiency of RP. Fourth, we only studied calcium oxalate composition BegoStones. The physical characteristics such as the crystalline structure and chemical properties of the fragments may vary between BegoStones and actual kidney stones. However, use of BegoStones allowed for a reproducible method for studying stone fragment migration. Another potential limitation is that RP could increase the renal pressure, which was not studied in our model. In a recent clinical series of 15 patients, we found that RP did not result in renal pelvic pressures > 30 mmHg [30]. Finally, our study used only one type of ureteroscope and access sheath, limiting the generalizability of findings to other instruments and different diameters or designs. It is likely that a larger sized UAS will occlude the ureter more completely and better prevent stone fragment migration. However, the larger size also increases the risk of urothelial or ischemic injury to the ureteral wall, increasing the risk of ureteral strictures.

## Conclusion

Retrograde pyeloperfusion demonstrated lower ureteral stone fragment migration compared to using an access sheath or keeping the ureter unoccupied. These benchtop results confirm our observations in the clinical setting. Future clinical studies could determine renal pelvic pressure when retrograde pyeloperfusion is employed and confirm the findings of this benchtop study. By reducing stone fragment migration, retrograde pyeloperfusion could contribute to shorter operative times, higher stone free rates, and better patient outcomes.

## Data Availability

No datasets were generated or analysed during the current study.

## Abbreviations

PCNL: Percutaneous Nephrolithotomy
RP: Retrograde Pyeloperfusion
UAS: Ureteral Access Sheath
UPJ: Ureteropelvic Junction
UVJ: Ureterovesical Junction
UIV: Ureters Crossing the Iliac Vessel
ECIRS: Endoscopic Combined Intrarenal Surgery
